# No-take marine reserves boost the resilience of commercial fish from the catastrophic effects of a volcanic eruption

**DOI:** 10.1371/journal.pone.0346563

**Published:** 2026-04-29

**Authors:** José Carlos Hernández, José Carlos Mendoza, Jesús M. Falcón, Sabrina Clemente, Pelayo Salinas, Natalí Denis-Lazzari, Carlos Sangil, Marc Balsalobre, David Martínez, Bernat Hereu, Alberto Brito

**Affiliations:** 1 Marine Community Ecology and Conservation, Departamento de Biología Animal, Edafología y Geología, Universidad de La Laguna, Tenerife, Canary Islands, Spain; 2 Centro Superior de Investigaciones Científicas - Instituto Español de Oceanografía en Canarias, Tenerife, Canary Islands, Spain; 3 Bioecomac, Departamento de Biología Animal, Edafología y Geología, Universidad de La Laguna, Tenerife, Canary Islands, Spain; 4 Estación Biológica Charles Darwin, Puerto Ayora, Santa Cruz Island, Ecuador; 5 Centro Superior de Investigaciones Científicas de Blanes, Barcelona, Spain; 6 Departamento de Botánica, Fisiología y Ecología, Universidad de La Laguna, Tenerife, Canary Islands, Spain; 7 Departamento de Ecología, Universitat de Barcelona, Barcelona, Spain; National Autonomous University of Mexico Institute of Geophysics: Universidad Nacional Autonoma de Mexico Instituto de Geofisica, MEXICO

## Abstract

Submarine volcanic eruptions are strong pulse disturbances that can cause abrupt mortality and long-lasting changes in marine communities. In October 2011, a submarine eruption off El Hierro (Canary Islands, Spain) generated a sulphurous plume that affected the Punta de La Restinga–Mar de Las Calmas Marine Reserve and surrounding coastal areas. Using a 25-year time series of fish community data, we assessed post-disturbance trajectories of commercial species across different protection levels within the impacted region. Resilience was analyzed as a sequential process, partitioned into resistance (initial biomass loss), recovery trajectory (temporal trend after the disturbance), and relative recovery compared with pre-eruption conditions. Because all sites were affected by the volcanic plume, our inference is restricted to comparative differences among protection categories rather than to the absolute effectiveness of protection. The no-take zone showed higher relative resistance and faster positive trajectories in total fish biomass than less-protected areas, indicating more favorable post-disturbance dynamics. However, eight years after the eruption, community structure had not fully returned to pre-eruption conditions in any protection level, reflecting contrasting recovery times among species with different life histories. Fast-growing species such as the parrotfish *Sparisoma cretense* recovered rapidly, whereas long-lived predators such as *Epinephelus marginatus* remained below their pre-disturbance biomass. Our results provide a long-term, community-level comparison of post-volcanic recovery within a marine reserve and highlight how protection status is associated with differences in recovery dynamics under a rare natural disturbance.

## Introduction

Submarine volcanic eruptions are rare pulse disturbances that can profoundly alter coastal ecosystems, yet their ecological consequences remain poorly understood over long temporal scales. Natural catastrophes are increasingly recognized as major drivers of marine community structure [1], but their effects are seldom evaluated using long-term datasets that allow the analysis of post-disturbance trajectories. On 10 October 2011, a submarine eruption off El Hierro (Canary Islands, Spain) generated an extensive sulphurous plume that rapidly covered the Punta de la Restinga–Mar de Las Calmas Marine Protected Area (PRMC-MR), causing extreme reductions in pH and oxygen and triggering recurrent fish mass mortalities [2–5]. This event represented a major ecological and socioeconomic disturbance for a community strongly dependent on small-scale fisheries and diving tourism [6]. Previous studies mainly described short-term responses of planktonic, microbial and benthic components [7–12], whereas long-term trajectories of fish assemblages remain largely unexplored.

Understanding how ecosystems respond to such disturbances is a central challenge in ecology because increasing environmental degradation can push systems towards undesired states with major biodiversity and socioeconomic losses [13–17]. For this reason, ecological resilience has become a key concept for conservation and management [18,19] and is embedded in major international policy frameworks such as the Aichi Biodiversity Targets, the Sustainable Development Goals and the Sendai Framework [20–22]. Despite the extensive theoretical development of resilience [23,24], empirical assessments that simultaneously address its multiple components and use long time series are still scarce.

In practice, resilience is not a single property but a dynamic process that includes resistance to disturbance, the trajectory of change after impact, and the extent of recovery relative to pre-disturbance conditions [25–28]. Because these components cannot be captured by a single metric [29], recent syntheses recommend explicitly defining the disturbance, the temporal framework and the ecological indicators used [30]. Here we follow these recommendations by operationally linking resistance, course and recovery to standardized community metrics (biomass, richness and diversity) in a long-term observational framework.

Marine protected areas (MPAs) are expected to influence post-disturbance dynamics by reducing local anthropogenic pressures and increasing biomass, size structure and functional diversity of exploited species [31–35]. These attributes have been hypothesized to enhance the capacity of ecosystems to absorb and reorganize after perturbations [36,37]. However, empirical evidence remains limited and largely restricted to coral reef systems, where resistance and recovery patterns in protected areas have shown contrasting results depending on the disturbance type and analytical framework [38–43]. Consequently, comparative assessments in other ecosystems and disturbance contexts are still needed.

Volcanic eruptions represent extreme pulse disturbances that can act as strong ecological filters, yet they are among the least studied events in marine systems. Existing studies have mainly focused on short-term recolonization processes or genetic recovery after historical eruptions [44–49]. The combination of a rare disturbance, a long pre-disturbance baseline and a spatial gradient of protection therefore provides a unique opportunity to examine how fish communities reorganize after a large-scale environmental shock.

Using a 25-year time series of fish assemblage data collected before and after the El Hierro eruption, we analyzed temporal changes across four protection levels (no-take, buffer, restricted and unprotected) within the impacted area. Specifically, we tested the hypotheses that (1) resistance, trajectory and recovery would differ among protection categories, and (2) these differences would be reflected in standardized community metrics and species-level responses. In general, we expect no-take zones to exhibit greater resistance than less protected areas, a more positive post-impact trajectory, and faster recovery in fish community descriptors. However, at the species level, we anticipate contrasting responses depending on life-history traits. Because all sites were affected by the volcanic plume, our inference is restricted to relative differences among protection levels rather than to the absolute effectiveness of protection. By explicitly partitioning resilience into its components in a long-term natural experiment, this study provides a transparent comparative assessment of post-disturbance dynamics and discusses its implications for marine spatial planning under increasing environmental variability.

## Materials and methods

### Study area: Nature and regime of the disturbance

**Fig 1:**
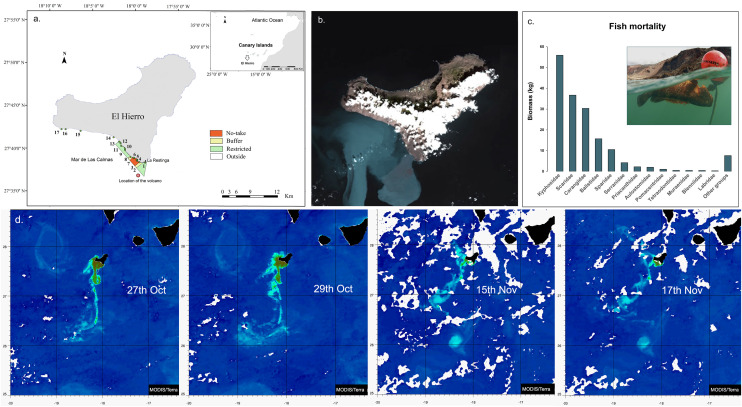
Sampling sites, sulfurous plume location and fish mortality registered. (a) Location of Punta Restinga – Mar de Las Calmas Marine Reserve of El Hierro Island in the Canary Islands showing the location of the volcano, La Restinga coastal town and the sampling sites: 1. La Restinga, 2. Playa de La Herradura, 3. Cueva de Los Frailes, 4. Punta de Los Frailes, 5. La Gabarra, 6. Roque Chico, 7. Roque de Naos, 8. Punta de Las Cañas, 9. Las Lapillas, 10. Cueva de El Diablo, 11. Tacorón, 12. Punta de Tifirabe, 13. Punta Lajas del Lance, 14. Punta de Linés, 15. Punta Los Mozos, 16. Laja de Orchilla, 17. Punta Palometa. (b) Satellite image (27^th^ of October 2011) of El Hierro Island showing the area affected by the volcanic eruption. Source: image taken from NASA Earth Observatory (public domain): http://earthobservatory.nasa.gov/ (c) Registered fish mortality that arrived at La Restinga port during 26^th^-29^th^ Oct. period; total biomasses are shown by families. (d) Images of 27^th^, 29^th^ Oct and 17^th^ Nov. Source: NASA MODIS/Terra, MOD09GA Surface Reflectance, 26 Feb 2026, accessed via NASA Worldview (public domain).

El Hierro Island is located on the western end of the Canarian Archipelago, and it is surrounded by oligotrophic and warm waters (19–25ºC SST), which are comparable to the open ocean subtropical gyres. A short basin surrounds the whole island, reaching great depths (3000m). Coastal submarine habitats are mostly characterized by a subtidal lava rocky bottom and a few sandy patches. The study area covers 7.46 km2 and represents a shallow rocky reef community that is up to 40 m deep. The island has been a UNESCO Biosphere Reserve since 2000 and one of the few examples worldwide where environmental sustainability is close to reality. Its wind-pumped hydropower station has succeeded in meeting 100% of the island demand for electricity exclusively with renewable energy for several days. A small-scale, sustainable fishery operated on the island with its main fishing harbor located in La Restinga, a coastal town on the southern tip of the island. Since 1996, a marine protected area was established on the western coast of the island. The marine reserve spans 1180 ha of the coast and encloses a 180 ha no-take zone where all fishing, harvesting and scuba-diving activities are forbidden apart from tunid fishing and scientific activities. There are two adjacent buffer zones that have fishing gear restrictions for moray traps and *Sarpa salpa* nets but where small-scale line fishing and controlled scuba diving are allowed. There is also a restricted fishing zone where small-scale fishing using traditional methods (no fish traps) and recreational line fishing from the shore are allowed based on previous authorization (Fig 1a).

The underwater volcanic eruption started on the 10^th^ of October 2011, 1.8 km south of La Restinga. The eruption was manifested by the presence of dead deep sea fishes (e.g., *Beryx splendens* and *Anthias anthias*) on the seawater surface and gray staining of the seawater column that soon turned green (Fig 1b). On the 23^rd^ of October, a sulfurous green plume was present on the shore until the 31^st^ of October and was followed by daily mass mortality of coastal fishes (Fig 1c) (S1 Fig). From the 1^st^ to the 18^th^ of November, the volcanic plume was again present on the shore, followed by another fish mass mortality event. On the 6^th^ of March 2012, the end of the volcanic eruption was declared. At the same time, the local government established a total fishing ban for the whole island until December 2012. In summary, the most intense plume was seen at the end of October and early November of 2011(Fig 1b and d). Although, there was intermitted sulfurous emissions from the volcano that affected the whole island, the sulfurous plume mainly affected the western coast of El Hierro Island for a total of 26 days. The spatiotemporal variability of the volcanic eruption plume was monitored by satellite, and a complete description can be found in Coca *et al*. (2014).

Most dead fish that arrived at La Restinga harbor during the first mortality event were identified and measured. Length (L)–weight (W) relationships for all recorded species were calculated using the Equation W = a × Lb to obtain the species biomass. *a* and *b* from the equation were obtained from available references for the Canary Islands or Fishbase (https://www.fishbase.se/search.php) (detailed information can be found in S2 Table). The total biomass recorded is summarized and classified by fish families in Fig 1c. These data can give us an estimation of the mortality magnitude but cannot be used as an absolute measure of fish mortality in the area. Fish mortality was accompanied by dramatic changes in oceanographic parameters [3]. Low oxygen concentrations together with low pH values recorded (with a minimum of 6.5 pH) in the volcanic plume contributed to the high mortality of invertebrates and fish observed in the area. Organisms died by a combination of anoxia and acidotic collapse of their metabolism. The drastic pH decreases due to volcanic CO_2_ emissions generated a large amount of H^+^ that saturated anhydrase carbonic acid, inhibited ATPase Na/K, occasioned blood acidosis and lowered hemoglobin affinity for O_2_. These combined metabolic shocks reduced the amount of O_2_ in the blood, causing suffocation in organisms that were later found dead or gasping at the surface of the water during the mortality events.

### Fish data collection

Fish communities have been surveyed at 17 sites through the marine reserve and outside since 1994, including before marine reserve enforcement started, and several years after in 1997, 1998, 2001, 2005, and 2008, including March 2012 (2012a) immediately after scientific diving was allowed by competent authorities and October 2012 (2012b), both coinciding with the fishing ban, and three (2014) and eight years (2019) after the eruption. A total of five sites in the no-take zone, two in the buffer zone, six in the restricted fishing zone and four outside the marine reserve (Fig 1a) were monitored. Fish surveys were conducted using a stationary visual census by means of scuba diving on rocky bottoms between 40 and 5 m depth. Specifically, we followed the point-count method as underwater visual census (UVC), in which the observer takes a position at the center of a circle (100 m^2^) and records the number and total length (± 2 cm) of individuals of each species [50]. At least six UVCs were conducted per site and subsequently averaged. Although all marine reserve zones were surveyed each year, adverse sea conditions occasionally prevented sampling at the same sites. Nevertheless, a minimum of three sites per marine reserve zone was consistently sampled each year. Fish species were classified by commercial, noncommercial and bycatch categories and by their mobility capacities (S2 Table). These categories were useful to test the effect of the eruption on the marine reserve fish resources that could not escape the volcanic eruption plume (sedentary and vagile). Species richness, Shannon diversity index and biomass (g· m^-2^) for fish species (calculations previously explained) were calculated for each surveyed site, marine reserve protection levels and surveyed years.

All fish data have been uploaded in Zenodo (S3 Link). All surveys conducted within the MPAs were carried out under permits issued by the *Consejería de Agricultura, Ganadería y Pesca* (Gobierno de Canarias) and by the *Dirección General de Recursos Pesqueros y Acuicultura* (Gobierno de España) (Authorizations 5/97–98, 6/05, 1/08, 2/12–14 and 6/19).

### Resilience partitioning and data analysis

Operationally, resilience was partitioned into (1) *resistance* or the ability of the system to absorb disturbance, (2) *course* or the trajectories of recovery, and (3) *recovery* as the ability of the system to recover to a pristine state (pre-perturbation state). Fish richness (number of species), fish Shannon diversity (H´) index and fish biomass were the three-metrics used to study the different components of resilience. These metrics were chosen because they are common metrics in most studies describing fish communities and may facilitate comparison among systems.

Different complex permutational ANOVA designs were applied to test each of the resilience components (resistance, course and recovery) on richness, diversity and total biomass fish data. A permutational MANOVA was also used to test species biomass composition. To test *resistance,* we used a two-way ANOVA with protection level and survey years as fixed factors. “Protection level” had four treatments: no-take, buffer, restricted and outside. For “Survey years”, we used the three previously sampled years before the volcanic eruption, 2001, 2005 and 2008, and 2012a, which was the first survey performed immediately after the volcanic eruption had finished. To test *course,* we used a similar two-way ANOVA, but this time, the “Survey years” factor included three surveys after the volcanic eruption had ended, 2012b, 2014 and 2019. Finally, to test *recovery,* we used the surveys in 2008, the last survey year before the eruption, and 2019, 8 years after the eruption.

PCO ordination was performed for each of the resilience components to visualize the fish community changes due to the factors “Protection level” or “Survey”. We identified the fish species mostly responsible for sample ordination using Pearson correlations between the first two PCO axes and fish species biomass, identified by a strength correlation with the axis of |r| > 0.4.

Sample-by-fish species matrices were pretreated using dispersion weighting because of schooling fish behavior that generated extreme abundance values. This pretreatment approach down weighs species whose counts are shown to be unreliable in replicates at the same site, with a high variance-to-mean ratio (dispersion index) over site replicates. Each species contribution was down weighed by the dispersion index, averaged over replicates [51]. The resemblance matrix was then calculated using Bray–Curtis similarity. All M/ANOVAs were based on the Bray–Curtis distances of the data, with p values obtained using 4000 permutations of the appropriate exchangeable units [52]. Significant terms in the full models were examined individually using appropriate *a posteriori* pairwise comparisons, also conducted by permutations. The software PRIMER7 & PERMANOVA [53] was used to perform all of the above statistical procedures.

We also compared the temporally lagged effects of underwater eruptions inside and outside the no-take area on the biomass of two essential species for coastal management (in the East Atlantic area) [54] and with contrasting life traits: the fast-growing, nonterritorial parrotfish *Sparisoma cretense* [55] and the slow-growing and territorial dusky grouper *Epinephelus marginatus* [56]. The magnitude of the effect was calculated by comparing prevolcanic biomass values with values after 5 months (2012a), 1 year (2012b), 3 years (2014) and 8 years (2019) from the detected fish mass mortalities.

## Results

### Disturbance magnitude: Fish mass mortality

**Fig 2:**
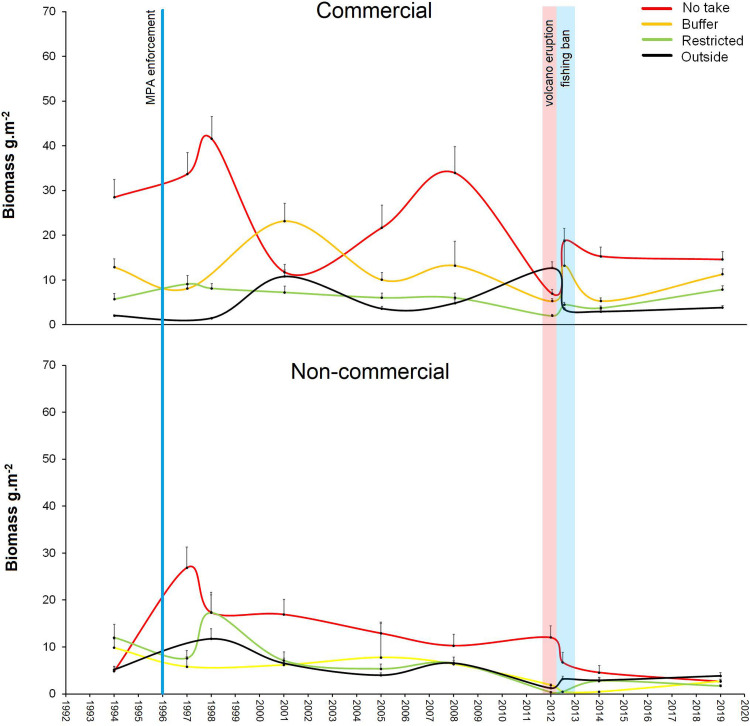
Historical trends of fish biomasses. Historical trends of commercial and non-comercial fish biomasses (mean + SD) by protection levels of Punta Restinga – Mar de Las Calmas Marine Reserve (El Hierro) during the surveyed years (1994, 1997, 1998, 2001, 2005, 2008, 2012a (March), 2012b (October), 2014 and 2019). Blue line show the enforcement year (1996); the red band shows the eruptive period (10^th^ of October – 6^th^ of March); and the blue band shows the fishing ban period (March – December 2012).

The volcanic eruption caused conspicuous mortality across 13 families of coastal fishes. The largest biomasses of dead individuals corresponded to Kyphosidae, Scaridae and Carangidae, followed by Balistidae, Sparidae and Serranidae (Fig 1c). These groups include key commercial species for the small-scale fleet of La Restinga, such as *Sparisoma cretense*, *Seriola* spp., *Balistes capriscus*, *Serranus atricauda* and *Epinephelus marginatus*. This taxonomic breadth indicates that the disturbance affected multiple trophic groups and mobility categories, providing a clear baseline for evaluating post-disturbance trajectories. Its impact is reflected in the decline in total biomass of both commercial and non-commercial species (Fig 2).

### Long-term biomass trajectories before and after the eruption

The 25-year time series revealed marked temporal variability in commercial fish biomass across protection levels (Fig 2). Prior to the eruption, biomass in the no-take zone fluctuated within a relatively wide range (10–40 g m ⁻ ²), whereas after the disturbance it remained consistently lower (5–18 g m ⁻ ²), indicating a system-wide reduction in biomass variability and magnitude. These oscillatory pre-disturbance dynamics were particularly evident in the no-take and buffer zones and were largely absent in the post-eruption period. Non-commercial species showed a similar but less pronounced pattern, with stronger temporal variability inside the no-take area.

### Relative resistance among protection levels

**Fig 3:**
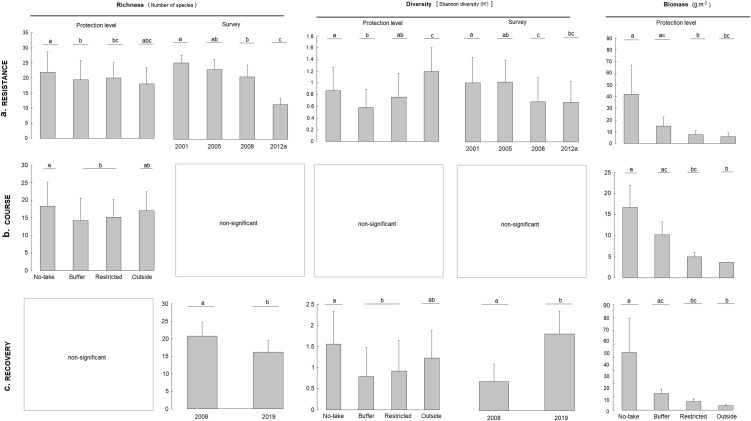
Bar plots of resilience metric partitioning. Significant effects of (a) *resistance*, (b) *course* and (c) *recovery* of commercial fish species to the underwater volcanic eruption using richness, diversity and biomass (average+SD) as metrics for the four protection levels and survey years. Letters a, b and c indicates the statistically pairwise comparisons between factor levels.

Immediately after the eruption (2012a), richness and diversity were significantly lower than in all pre-eruption surveys, indicating a strong initial impact across the study area. However, the magnitude of this response differed among protection levels. Species richness was consistently higher in the no-take zone than in buffer and restricted areas, while total biomass was also greatest inside the no-take zone and significantly lower in restricted and outside areas (Table 1; Fig 3a). Diversity followed a similar pattern, with the no-take zone differing from buffer and outside areas. These differences represent relative contrasts among protection levels within the impacted area rather than absolute resistance to the disturbance.

**Table 1:** Results of the two-way permutational analyses of variance (PerANOVA) of richness, diversity and total biomass of commercial fish species testing the resistance of the fish community across protection levels of the marine reserve and the survey years. Multiple surveys before the eruption (2001, 2005, 2008) vs. survey immediate after the eruption (2012a). PL: protection level; S: survey year.

BIOMASS					
Source	df	MS	Pseudo-F	P(perm)	Unique perms
Protection level	3	5202.9	3.7964	**0.0027**	3986
Survey	3	2364.5	1.7253	0.1095	3992
PLxS	9	1217.3	0.88824	0.6186	3988
Res	25	1370.5			
Total	40				
**Protection level Pairwise**					
t					
No-take vs Restricted			3.1859	**0.0005**	3989
No-take vs Outside			1.9041	**0.0182**	3993
No-take vs Buffer			1.1544	0.2769	3991
Restricted vs Outside			0.52733	0.8008	3990
Restricted vs Buffer			2.1493	**0.0315**	3988
Outside vs Buffer			1.162	0.2764	3997
**RICHNESS**					
**Source**	**df**	**MS**	**Pseudo-F**	**P(perm)**	**Unique perms**
Protection level	3	34.832	4.8499	**0.0075**	3991
Survey	3	317.69	44.234	**0.0002**	3992
PLxS	9	6.7183	0.93544	0.5104	3991
Res	25	7.182			
Total	40				
**Protection level Pairwise**					
t					
No-take vs Restricted			3.9784	**0.0007**	3971
No-take vs Outside			1.9906	0.0742	3975
No-take vs Buffer			3.1002	**0.0075**	3971
Restricted vs Outside			0.46191	0.6568	3958
Restricted vs Buffer			0.70304	0.4869	3969
Outside vs Buffer			0.022263	0.9813	3967
**Survey Pairwise**					
t					
2001 vs 2005			1.9894	0.0677	3960
2001 vs 2008			4.2199	**0.0005**	3964
2001 vs 2012a			13.94	**0.0002**	3969
2005 vs 2008			1.6332	0.1285	3979
2005 vs 2012a			8.2668	**0.0002**	3977
2008 vs 2012a			7.1458	**0.0002**	3973
**DIVERSITY**					
Source	df	MS	Pseudo-F	P(perm)	Unique perms
Protection level	3	0.6966	6.5191	**0.0015**	3991
Survey	3	0.48407	4.5302	**0.0115**	3989
PLxS	9	0.13996	1.3099	0.2819	3992
Res	25	0.10685			
Total	40				
**Protection level Pairwise**					
t					
No-take vs Restricted			1.4245	0.1705	3966
No-take vs Outside			2.7368	**0.014**	3973
No-take vs Buffer			2.3404	**0.033**	3971
Restricted vs Outside			3.3317	**0.0047**	3962
Restricted vs Buffer			0.73232	0.4696	3971
Outside vs Buffer			4.3665	**0.0032**	3979
**Survey Pairwise**					
t					
2001 vs 2005			0.56631	0.5701	3965
2001 vs 2008			3.0107	**0.0087**	3976
2001 vs 2012a			2.5235	**0.0305**	3972
2005 vs 2008			2.4126	**0.0267**	3968
2005 vs 2012a			2.0138	0.0737	3959
2008 vs 2012a			0.6781	0.4979	3968

### Post-disturbance trajectories (course)

During the post-eruption period (2012b–2019), recovery trajectories differed among protection levels. Richness and total biomass remained significantly higher in the no-take zone than in restricted and outside areas, while diversity showed no clear protection-level effect (Table 2; Fig 3b). Temporal comparisons indicated progressive increases in biomass, particularly inside the no-take zone, whereas outside areas showed slower and more limited changes. These patterns reflect divergent recovery trajectories rather than complete recovery of the pre-disturbance state.

**Table 2:** Results of the two-way permutational analyses of variance (PerANOVA) of richness, diversity and total biomass of commercial fish species testing the resilience of the fish community across protection levels of the marine reserve and the survey years. Multiple surveys one year after the eruption (2012b, 2014, 2019). PL: protection level; S: survey year.

RICHNESS					
Source	df	MS	Pseudo-F	P(perm)	Unique perms
Protection level	3	28.289	3.7033	**0.0305**	3994
Survey	2	4.1548	0.5439	0.5891	3990
PLxS	6	2.3056	0.30182	0.9195	3990
Res	18	7.6389			
Total	29				
**Protection level Pairwise**					
t					
No-take vs Outside			0.85585	0.4049	3934
No-take vs Buffer			2.8868	**0.012**	3938
No-take vs Restricted			2.3938	**0.0335**	3931
Outside vs Buffer			1.6435	0.1417	3584
Outside vs Restricted			1.2702	0.2219	3333
Buffer vs Restricted			0.92582	0.4079	568
**DIVERSITY**					
Source	df	MS	Pseudo-F	P(perm)	Unique perms
Protection level	3	0.56856	2.0923	0.1417	3997
Survey	2	0.43254	1.5917	0.2407	3993
PLxS	6	0.08197	0.30164	0.9263	3995
Res	18	0.27174			
Total	29				
**BIOMASS**					
Source	df	MS	Pseudo-F	P(perm)	Unique perms
Protection level	3	4500.9	5.6064	**0.003**	3993
Survey	2	865.6	1.0782	0.3657	3995
PLxS	6	369.49	0.46024	0.9138	3983
Res	18	802.82			
Total	29				
**Protection level Pairwise**					
t					
No-take vs Outside			3.3755	**0.004**	3991
No-take vs Buffer			1.0808	0.3052	3993
No-take vs Restricted			2.6811	**0.0122**	3981
Outside vs Buffer			2.7716	**0.0207**	3909
Outside vs Restricted			0.9211	0.4024	3911
Buffer vs Restricted			1.8593	0.0875	3916

### Long-term recovery patterns

Eight years after the eruption (2019), total biomass still depended on protection level, with the highest values in the no-take zone and significantly lower values in restricted and outside areas (Table 3; Fig 3c). Species richness was lower in 2019 than in the last pre-eruption survey (2008), indicating incomplete recovery at the assemblage level. In contrast, diversity increased between 2008 and 2019, suggesting a reorganization of community structure rather than a simple return to the previous state.

**Table 3:** Results of the two-way permutational analyses of variance (PerANOVA) of richness, diversity and total biomass of commercial fish species testing the recovery of the fish community across protection levels of the marine reserve and the survey years. Last survey before the eruption (2008) vs. eight years after the eruption (2019). PL: protection level; S: survey year.

RICHNESS					
Source	df	MS	Pseudo-F	P(perm)	Unique perms
Protection level	3	29.745	2.6426	0.0895	3991
Survey	1	105.18	9.3442	**0.007**	3977
PLxS	3	6.7077	0.59593	0.6286	3994
Res	14	11.256			
Total	21				
**Survey Pairwise**					
t					
2008 vs 2019			3.0568	**0.0077**	3978
**DIVERSITY**					
Source	df	MS	Pseudo-F	P(perm)	Unique perms
Protection level	3	0.60591	3.5185	**0.0382**	3993
Survey	1	5.9641	34.633	**0.0002**	3955
PLxS	3	0.088957	0.51657	0.6741	3992
Res	14	0.17221			
Total	21				
**Protection level Pairwise**					
t					
No-take vs Outside			0.96732	0.3574	3938
No-take vs Buffer			3.2959	**0.013**	3813
No-take vs Restricted			2.5933	**0.029**	3950
Outside vs Buffer			1.442	0.2127	2439
Outside vs Restricted			0.85579	0.4266	3360
Buffer vs Restricted			1.2443	0.2759	2497
**Survey Pairwise**					
t					
2008 vs 2019			5.885	**0.0005**	3971
**BIOMASS**					
Source	df	MS	Pseudo-F	P(perm)	Unique perm
Protection level	3	4166.6	4.2618	**0.0025**	3993
Survey	1	555.73	0.56842	0.6626	3987
PLxS	3	545.81	0.55827	0.8548	3990
Res	14	977.68			
Total	21				
**Protection level Pairwise**					
t					
No-take vs Outside			2.6016	**0.0025**	3974
No-take vs Buffer			0.95008	0.4514	3842
No-take vs Restricted			2.0621	**0.0295**	3974
Outside vs Buffer			2.5341	**0.0245**	2427
Outside vs Restricted			1.6639	0.119	3430
Buffer vs Restricted			2.1931	0.077	2431

### Fish community reassembly

**Fig 4:**
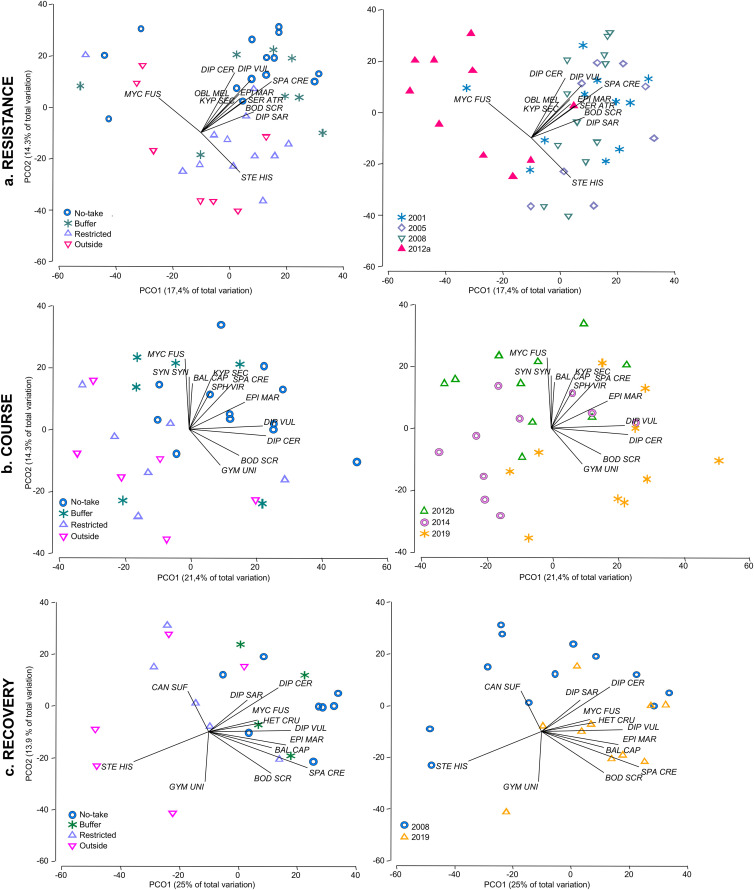
Ordination plots of fish assemblages. Biplot of the first two Principal Component axes of the Principal Component analysis (PCO) examining the effect of the factor “protection level” and “survey” on assemblages of commercial fish species. Vectors represent correlations between the two first PCO axes and fish species biomasses, identified by a strength Pearson correlation with the axis of |r| > 0.4. Abbreviations are BAL CAP (*Balistes capriscus*), BOD SCR (*Bodianus scrofa*), CAN SUF (*Canthidermis suflamen*), DIP CER (*Diplodus cervinus*), DIP SAR (*Diplodus sargus*), DIP VUL (*Diplodus vulgaris*), EPI MAR (*Epinephelus marginatus*), GYM UNI (*Gymnotorax unicolor*), HET CRU (*Heteropriacanthus cruentatus*), KYP SEC (*Kyphosus sectatrix*), OBL MEL (*Oblada melanura*), SER ATR (*Serranus atricauda*), MYC FUS (*Mycteroperca fusca*), SPA CRE (*Sparisoma cretense*), SPH VIR (*Sphyraena viridensis*), STE HIS (*Stephanolepis hispidus*) and SYN SYN (*Synodus synodus*).

Multivariate analyses detected significant effects of both protection level and survey year for the three resilience components (Table 4). Immediately after the eruption, fish assemblages differed from all pre-disturbance surveys and were characterized by reduced biomass of the dominant pre-eruption species. Across the time series, assemblages in the no-take and buffer zones were associated with higher biomass of *Sparisoma cretense*, *Diplodus* spp., *Epinephelus marginatus*, *Serranus atricauda* and *Bodianus scrofa*. In contrast, outside areas were consistently separated in ordination space. Post-eruption surveys showed a progressive shift towards assemblages dominated by these species, particularly inside the no-take zone, whereas restricted and outside areas remained more variable (Fig 4).

**Table 4:** Results of the two-way permutational analyses of variance (PERMANOVA) for the fish community biomasses composition testing the resistance, course and recovery the fish community across protection levels of the marine reserve and the survey years. PL: protection level; S: survey year.

RESISTANCE					
Source	df	MS	Pseudo-F	P(perm)	Unique perms
Protection level	3	5206.8	2.1177	**0.0007**	3982
Survey	3	5848.5	2.3786	**0.0002**	3980
PLxS	9	2334.4	0.94941	0.6371	3972
Res	25	2458.8			
Total	40				
**Protection level Pairwise**					
t					
No-take vs Restricted			1.7019	**0.0005**	3986
No-take vs Outside			1.8198	**0.0015**	3987
No-take vs Buffer			1.1644	0.19	3989
Restricted vs Outside			1.0613	0.3487	3987
Restricted vs Buffer			1.2078	0.1495	3995
Outside vs Buffer			1.5977	**0.0282**	3988
**Survey Pairwise**					
t					
2001 vs 2005			1.2083	0.1665	3990
2001 vs 2008			1.0159	0.4304	3990
2001 vs 2012a			1.6124	**0.003**	3982
2005 vs 2008			1.0276	0.4174	3987
2005 vs 2012a			1.6827	**0.0102**	3993
2008 vs 2012a			1.9611	**0.0005**	3988
**RESILIENCE**					
Source	df	MS	Pseudo-F	P(perm)	Unique perms
Protection level	3	3683.6	2.0215	**0.003**	3985
Survey	2	5059.3	2.7764	**0.0007**	3991
PLxS	6	1493	0.81933	0.8743	3977
Res	18	1822.3			
Total	29				
**Protection level Pairwise**					
t					
No-take vs Outside			1.7297	**0.0047**	3992
No-take vs Buffer			1.5495	**0.0212**	3990
No-take vs Restricted			1.5963	**0.0157**	3986
Outside vs Buffer			1.2749	0.1782	3901
Outsidevs Restricted			0.80693	0.7208	3903
Buffer vs Restricted			1.1201	0.2917	3898
**Survey Pairwise**					
t					
2012b vs 2014			1.3082	0.0677	3989
2012b vs 2019			1.942	**0.003**	3986
2014 vs 2019			1.6679	**0.0077**	3987
**RECOVERY**					
Source	df	MS	Pseudo-F	P(perm)	Unique perms
Protection level	3	4161.4	2.0891	**0.0015**	3980
Survey	1	5231.4	2.6263	**0.003**	3992
PLxS	3	1798.7	0.90299	0.6438	3978
Res	14	1991.9			
Total	21				
**Protection level Pairwise**					
t					
No-take vs Outside			2.1306	**0.0015**	3973
No-take vs Buffer			1.1142	0.2974	3852
No-take vs Restricted			1.4645	**0.034**	3979
Outside vs Buffer			1.3935	0.1165	2493
Outside vs Restricted			1.1402	0.2672	3429
Buffer vs Restricted			1.0815	0.3747	2429
**Survey Pairwise**					
t					
2008 vs 2019			1.6206	0.0022	3990

### Species-specific responses

**Fig 5:**
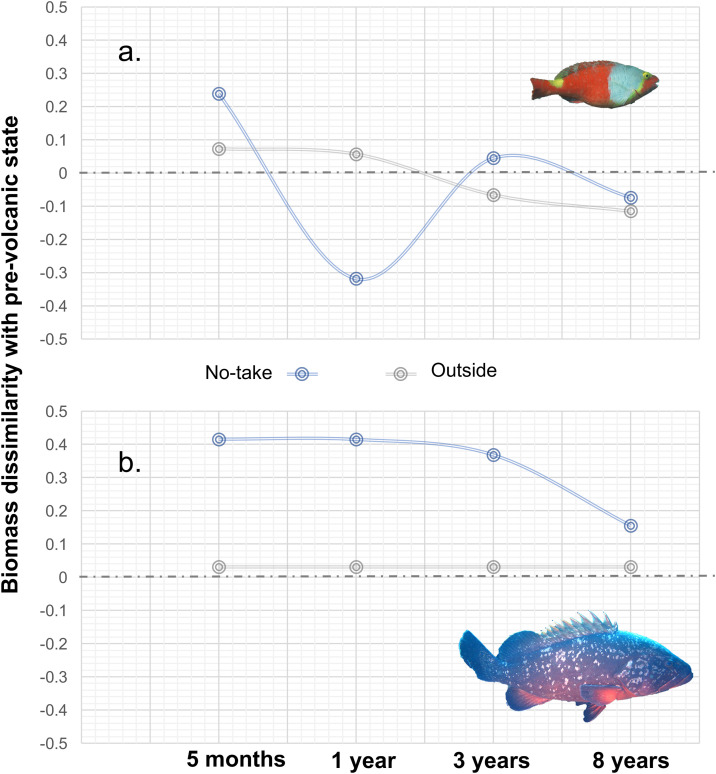
Temporal lagged effect of the volcanic eruption on the fish biomass of the species (a) *Sparisoma cretense* and (b) *Epinephelus marginatus* at the no-take and outside zones. Comparisons are made between the surveyed year 2008, just before the eruption took place, and after 5 months (2012a), 1 year (2012b), 3 (2014) and 8 years (2019) from the first fish mass mortality. The zero-grey dashed line shows no dissimilarity with pre-disturbance state (2008), positive and negative values shows a decline or an increase, respectively, in the pre-volcanic fish biomass values.

The two focal commercial species displayed contrasting trajectories inside the no-take zone. *Sparisoma cretense* showed a rapid increase in biomass, exceeding pre-disturbance values within one year, whereas *Epinephelus marginatus* remained below its pre-eruption biomass after eight years, although it exhibited a positive trend in the last surveys (Fig 5). These differences are consistent with their contrasting life-history traits and contributed to the incomplete recovery of total biomass and richness at the assemblage level.

## Discussion

This study documents the short- and medium-term responses of a subtropical rocky-reef fish community to a rare pulse disturbance and provides a comparative assessment of recovery trajectories across protection levels within the impacted area. A post-eruption sulphurous plume generated recurrent fish mass mortalities along the western coast of El Hierro, producing a system-wide ecological impact. By combining this natural perturbation with a 25-year time series, our results reveal consistent differences among protection categories in resistance, post-disturbance trajectories, and partial recovery of key commercial fish metrics. However, because all sampling sites were affected by the volcanic plume, these patterns should be interpreted as relative differences within the disturbed system rather than as a direct test of the absolute effectiveness of protection or of full recovery to a pre-disturbance state.

Partitioning resilience into resistance, course, and recovery, and linking these components to standardized community metrics, allowed a sequential and operational interpretation of a complex ecological process [30]. Immediately after the disturbance, species richness and diversity declined across protection levels, indicating a strong system-wide impact consistent with the observed mass mortalities. In contrast, total commercial fish biomass showed higher resistance and a more positive post-disturbance trajectory in the no-take and, to a lesser extent, in the buffer zone. Eight years after the eruption, biomass and diversity in these zones showed clear signs of recovery relative to the other protection levels, although pre-eruption values were not fully reached. The increase in diversity despite reduced richness suggests a more even distribution of biomass among fewer species, reflecting a community readjustment rather than full recovery.

Changes in community structure further support this interpretation. The immediate post-eruption assemblage was dominated by highly mobile species, particularly *Mycteroperca fusca*, highlighting the importance of mobility and the ability to escape the plume. In subsequent years, the no-take and buffer zones were consistently associated with higher biomass of commercially important species such as *Sparisoma cretense*, *Diplodus* spp., *Epinephelus marginatus*, *Serranus atricauda*, and *Bodianus scrofa*. These trajectories likely reflect the combined influence of protection status, spatial variability in disturbance intensity, and pre-existing ecological differences among sites.

Species-specific responses emphasize the role of life-history traits in post-disturbance dynamics. The fast-growing parrotfish *Sparisoma cretense* rapidly returned to, and even exceeded, its pre-eruption biomass within one year in the no-take zone, whereas the slow-growing and territorial dusky grouper *Epinephelus marginatus* showed a much slower trajectory and had not reached pre-disturbance biomass after eight years. Differences in age at maturity [57,58], growth rates, and home-range size [59] provide a plausible explanation for these contrasting recovery times and suggest that spillover from adjacent areas may also have contributed to the rapid recovery of mobile species.

The comparative framework used here is consistent with previous empirical studies showing that protected areas can influence post-disturbance trajectories, although the direction and magnitude of these effects vary among systems and disturbance types [39–43]. Our results extend this body of work to a subtropical rocky-reef ecosystem affected by underwater volcanism, a disturbance that has rarely been examined over long temporal scales. The persistence of macroalgal habitats after the eruption [10], together with the temporary increase in nutrient availability [3], likely facilitated the relatively rapid response of herbivorous and omnivorous fishes.

Several mechanisms may contribute to the comparatively higher resistance and faster biomass trajectories observed in the most protected zones. Long-term protection is typically associated with greater standing biomass and size structure, as well as increased functional diversity [60–64], which can buffer the demographic effects of sudden mortality events [65]. Functional redundancy and trophic interactions may also enhance the capacity of communities to reorganize after disturbance [66,67], and processes such as herbivory and portfolio effects have been proposed as key mechanisms in other systems [38,68]. Nevertheless, given the observational nature of our study and the spatial variability in plume exposure, these mechanisms should be regarded as plausible but not demonstrable causal processes.

From a management perspective, our results provide comparative evidence that long-established no-take areas can be associated with more favorable post-disturbance trajectories for commercially important fish biomass. At the same time, the incomplete recovery observed after eight years indicates that a single protected area may not be sufficient to buffer the effects of large-scale catastrophes. This supports previous calls to incorporate catastrophe planning into MPA design [69] and highlights the importance of networks of protected areas for spreading risk and enhancing regional resilience [70].

The existence of a detailed pre-disturbance baseline makes this system a valuable natural experiment for understanding community reassembly after extreme events. By explicitly defining resilience components, using standardized metrics, and adopting a cautious inferential framework, this study contributes to a more operational and comparable assessment of resilience in marine ecosystems. Rather than demonstrating the absolute effectiveness of protection, our results show that protection status is associated with divergent recovery trajectories within a disturbed seascape and underscore the importance of long-term monitoring for detecting and interpreting these dynamics.

## Conclusions

This long-term natural experiment shows that a submarine volcanic eruption produced a strong system-wide impact on coastal fish assemblages, with an immediate decline in richness and diversity across all protection levels and a recovery process that remained incomplete after eight years. By partitioning resilience into resistance, course and recovery, we demonstrate that post-disturbance dynamics followed divergent trajectories within the disturbed seascape, with no-take and buffer zones consistently maintaining higher commercial biomass and more positive temporal trends than less protected areas. These patterns represent relative differences rather than evidence of full recovery or absolute reserve effectiveness.

The contrasting responses of species with different life-history traits indicate that recovery rates are strongly trait-dependent, and the increase in diversity despite reduced richness reflects community reorganization instead of a simple return to the pre-eruption state. The availability of a long pre-disturbance baseline highlights the critical importance of sustained monitoring for detecting and interpreting the ecological consequences of rare catastrophic events.

From a management perspective, our results suggest that long-term protection can be associated with more favorable biomass trajectories after large-scale disturbances, but also that single reserves are unlikely to buffer such events on their own. Incorporating catastrophic disturbances into marine spatial planning and developing networks of protected areas will therefore be essential to enhance resilience under increasing environmental variability.

## Supporting information

S1 Fig Fish pictures taken during the field surveys. Pictures taken during the first dives after the volcanic eruption. (a) Dusky grouper *Epinephelus marginatus* found dead next to the marine reserve buoy during the second fish mass mortality; (b) *E. marginatus* bones found in the littoral caves of the no-take area during the first survey after the eruption; (c) Islands grouper *Mycteroperca rubra* at the Laja de Orchilla site, edge of the affected area; (d) Parrofish *Sparisoma cretense* juveniles and small adults registered during the second survey after the eruption (2012b); (e) *Diplodus sargus* and *D. vulgaris* juveniles during the second survey (2012b); (f) Juveniles of *Diplodus cervinus* during the second survey (2012b). All pictures were taken by the authors (JCH and PS).
(PDF)

S2 Table Fish species sampled. List of the fish species sampled in the shallow rocky sublitoral community of El Hierro Island (1996–2019), specifying authority, commercial value, mobility and average biomass.
(DOCX)
