# Supplementary figures and images for: No-take marine reserves boost the resilience of commercial fish from the catastrophic effects of a volcanic eruption
Source: PLoS One. 2026 Apr 29;21(4):e0346563. doi: 10.1371/journal.pone.0346563 (PMC13127932; doi:10.1371/journal.pone.0346563)

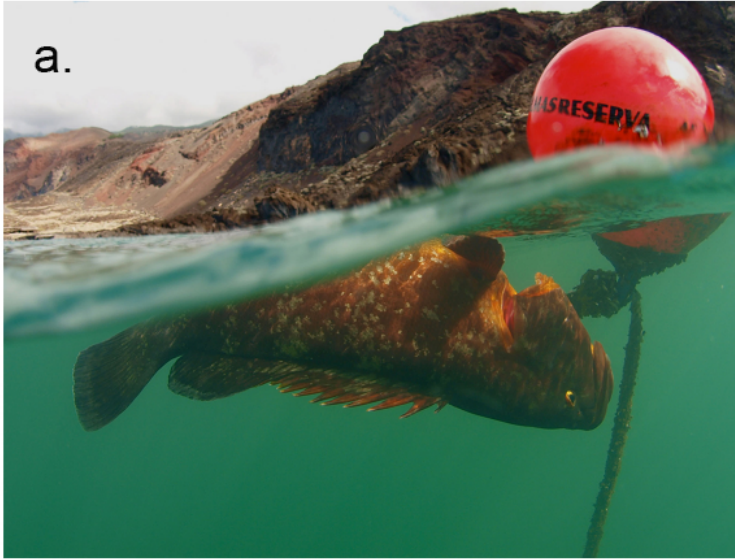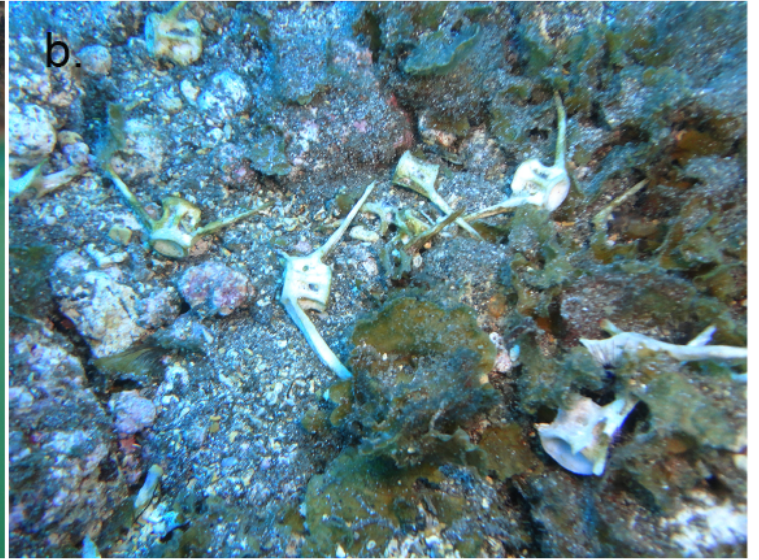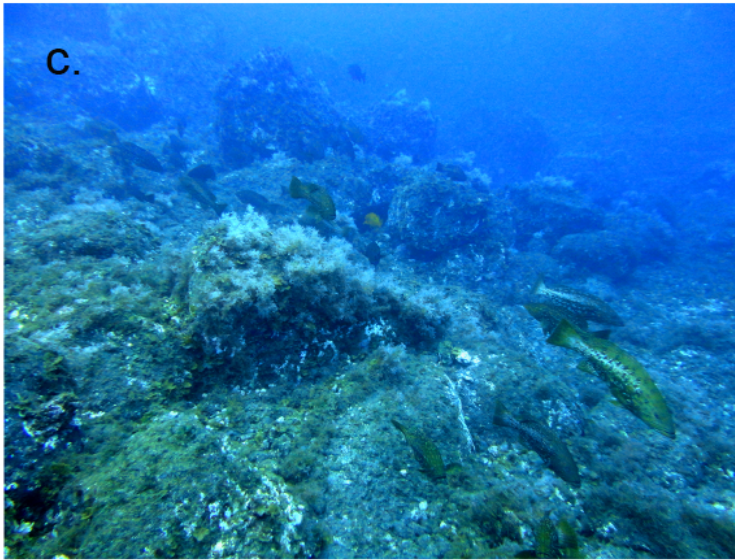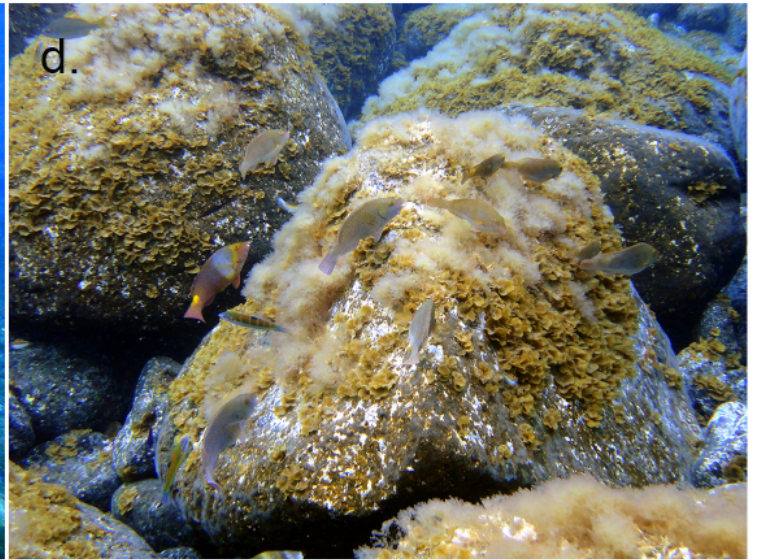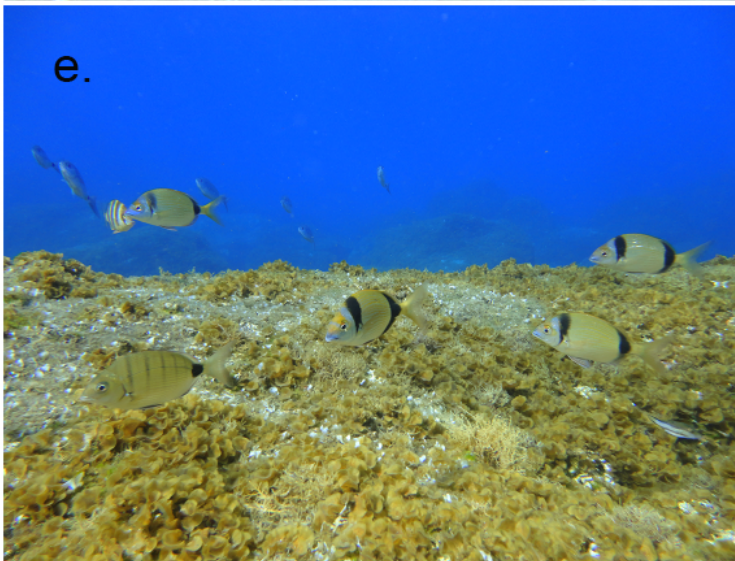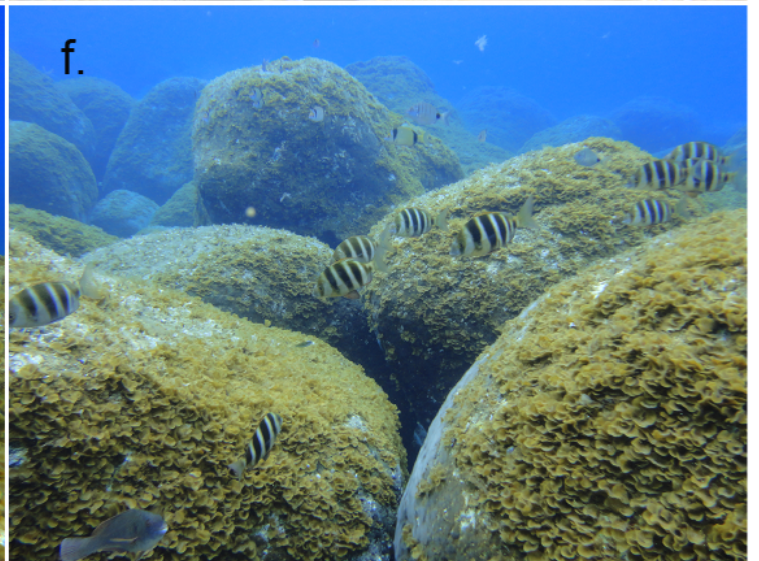

Supplement: S1 Fig — Pictures taken during the first dives after the volcanic eruption. (a) Dusky grouper Epinephelus marginatus found dead next to the marine reserve buoy during the second fish mass mortality; (b) E. marginatus bones found in the littoral caves of the no-take area during the first survey after the eruption; (c) Islands grouper Mycteroperca rubra at the Laja de Orchilla site, edge of the affected area; (d) Parrofish Sparisoma cretense juveniles and small adults registered during the second survey after the eruption (2012b); (e) Diplodus sargus and D. vulgaris juveniles during the second survey (2012b); (f) Juveniles of Diplodus cervinus during the second survey (2012b). All pictures were taken by the authors (JCH and PS). (PDF) [file pone.0346563.s001.pdf]
