# Supplementary material for: No-take marine reserves boost the resilience of commercial fish from the catastrophic effects of a volcanic eruption
Source: PLoS One. 2026 Apr 29;21(4):e0346563. doi: 10.1371/journal.pone.0346563 (PMC13127932; doi:10.1371/journal.pone.0346563)
Supplement: S2 Table — List of the fish species sampled in the shallow rocky sublitoral community of El Hierro Island (1996–2019), specifying authority, commercial value, mobility and average biomass. (DOCX) [file pone.0346563.s002.docx]

| **Family** | **Species** | **Authority** | **Commercial**  **value** | **Mobility** | **Average** |
| --- | --- | --- | --- | --- | --- |
|  |  |  |  |  | **Biomass (g.m^-2^)** |
| Apogonidae | *Apogon imberbis* | (Linnaeus, 1758) | non commercial | vagile | 0.001 |
| Atherinidae | *Atherina presbyter* | (Cuvier, 1829) | commercial | vagile | 0.001 |
| Aulostomidae | *Aulostomus strigosus* | (Wheeler, 1955) | non commercial | vagile | 1.014 |
| Balistidae | *Balistes capriscus* | (Gmelin, 1789) | commercial | very vagile | 0.073 |
|  | *Canthidermis sufflamen* | (Mitchill, 1815) | commercial | very vagile | 0.158 |
| Belonidae | *Belone belone* | (Linnaeus, 1760) | non commercial | very vagile | < 0.001 |
| Blenniidae | *Ophioblennius atlanticus* | (Valenciennes, 1836) | non commercial | sedentary | 0.001 |
|  | *Parablennius pilicornis* | (Cuvier, 1829) | non commercial | sedentary | < 0.001 |
| Bothidae | *Bothus podas* | (Delaroche, 1809) | bycatch | sedentary | < 0.001 |
| Carangidae | *Pseudocaranx dentex* | (Bloch & Schneider, 1801) | commercial | very vagile | 0.089 |
|  | *Caranx crysos* | (Mitchill, 1815) | commercial | very vagile | 0.011 |
|  | *Caranx latus* | (Agassiz, 1831) | commercial | very vagile | 0.002 |
|  | *Seriola dumerilii* | (Risso, 1810) | commercial | very vagile | 0.225 |
|  | *Seriola fasciata* | (Bloch, 1793) | commercial | very vagile | 0.007 |
|  | *Seriola rivoliana* | (Valenciennes, 1833) | commercial | very vagile | 0.047 |
|  | *Trachinotus ovatus* | (Linnaeus, 1758) | commercial | vagile | 0.781 |
|  | *Caranx lugubris* | (Poey, 1860) | commercial | very vagile | 0.005 |
| Clupeidae | *Sardina pilchardus* | (Walbaum, 1792) | commercial | very vagile | 0.049 |
|  | *Sardinella aurita* | (Valenciennes, 1847) | commercial | very vagile | 0.033 |
| Congridae | *Heteroconger longissimus* | (Günther, 1870) | non commercial | sedentary | 0.165 |
| Dasyatidae | *Bathytoshia centroura* | (Mitchill, 1815) | bycatch | vagile | 0.017 |
|  | *Dasyatis pastinaca* | (Linnaeus, 1758) | bycatch | vagile | 0.009 |
|  | *Taeniura grabata* | (Geoffroy Saint-Hilaire, 1817) | bycatch | vagile | 0.123 |
| Diodontidae | *Chilomycterus reticulatus* | (Linnaeus, 1758) | non commercial | vagile | 0.105 |
| Gobiesocidae | *Lepadogaster candollei* | (Risso, 1810) | non commercial | sedentary | < 0.001 |
|  | *Diplecogaster ctenocrypta* | (Briggs, 1955) | non commercial | sedentary | < 0.001 |
| Gobiidae | *Gnatholepis thomsoni* | (Jordan, 1904) | non commercial | sedentary | 0.001 |
| Gymnuridae | *Gymnura altavela* | (Linnaeus, 1758) | bycatch | vagile | 0.089 |
| Haemulidae | *Pomadasys incisus* | (Bowdich, 1825) | commercial | vagile | 0.027 |
| Kyphosidae | *Kyphosus sectatrix* | (Linnaeus, 1758) | commercial | very vagile vagile | 0.490 |
| Labridae | *Symphodus trutta* | (Lowe, 1834) | bycatch | vagile | 0.001 |
|  | *Coris julis* | (Linnaeus, 1758) | non commercial | vagile | 0.003 |
|  | *Thalassoma pavo* | (Linnaeus, 1758) | non commercial | vagile | 0.841 |
|  | *Xyrichtys novacula* | (Linnaeus, 1758) | bycatch | vagile | < 0.001 |
|  | *Bodianus scrofa* | (Valenciennes, 1839) | commercial | vagile | 0.264 |
| Monacanthidae | *Aluterus scriptus* | (Osbeck, 1765) | bycatch | vagile | 0.102 |
|  | *Stephanolepis hispidus* | (Linnaeus, 1766) | commercial | vagile | 0.004 |
| Mugilidae | *Chelon labrosus* | (Risso, 1827) | commercial | vagile | 0.045 |
|  | *Mugil cephalus* | (Linnaeus, 1758) | commercial | vagile | 0.054 |
| Mullidae | *Mullus surmuletus* | (Linnaeus, 1758) | commercial | vagile | 0.001 |
|  | *Mulloidichthys martinicus* | (Cuvier, 1829) | commercial | vagile | < 0.001 |
| Muraenidae | *Enchelycore anatina* | (Lowe, 1838) | commercial | sedentary | 0.005 |
|  | *Gymnothorax miliaris* | (Kaup, 1856) | commercial | sedentary | 0.003 |
|  | *Gymnothorax unicolor* | (Delaroche, 1809) | commercial | sedentary | 0.052 |
|  | *Muraena augusti* | (Kaup, 1856) | commercial | sedentary | 0.055 |
| Myliobatidae | *Myliobatis aquila* | (Linnaeus, 1758) | bycatch | vagile | 0.010 |
|  | *Pteromylaeus bovinus* | (Geoffroy Saint-Hilaire, 1817) | bycatch | vagile | 0.013 |
| Ophichthidae | *Myrichthys pardalis* | (Valenciennes, 1839) | non commercial | vagile | < 0.001 |
| Pomacentridae | *Chromis limbata* | (Valenciennes, 1833) | non commercial | vagile | 0.441 |
|  | *Similiparma lurida* | (Cuvier, 1830) | non commercial | vagile | 0.131 |
| Priacanthidae | *Heteropriacanthus cruentatus* | (Lacepède, 1801) | commercial | vagile | 0.052 |
| Scaridae | *Sparisoma cretense* | (Linnaeus, 1758) | commercial | vagile | 1.435 |
| Sciaenidae | *Sciaena umbra* | (Linnaeus, 1758) | commercial | vagile | 0.016 |
| Scombridae | *Katsuwonus pelamis* | (Linnaeus, 1758) | commercial | very vagile | 0.112 |
|  | *Sarda sarda* | (Bloch, 1793) | commercial | very vagile | 0.009 |
|  | *Scomber colias* | (Gmelin, 1789) | commercial | vagile | 0.045 |
| Scorpaenidae | *Scorpaena maderensis* | (Valenciennes, 1833) | non commercial | sedentary | 0.007 |
|  | *Scorpaena porcus* | (Linnaeus, 1758) | commercial | sedentary | 0.001 |
|  | *Scorpaena scrofa* | (Linnaeus, 1758) | commercial | sedentary | < 0.001 |
| Serranidae | *Anthias anthias* | (Linnaeus, 1758) | non commercial | vagile | < 0.001 |
|  | *Epinephelus costae* | (Steindachner, 1878) | commercial | vagile | < 0.001 |
|  | *Epinephelus marginatus* | (Lowe, 1834) | commercial | vagile | 0.855 |
|  | *Serranus atricauda* | (Günther, 1874) | commercial | vagile | 0.089 |
|  | *Serranus cabrilla* | (Linnaeus, 1758) | commercial | vagile | < 0.001 |
|  | *Serranus papilionaceus* | (Linnaeus, 1758) | commercial | vagile | < 0.001 |
|  | *Mycteroperca fusca* | (Lowe, 1838) | commercial | vagile | 0.162 |
| Sparidae | *Sarpa salpa* | (Linnaeus, 1758) | commercial | vagile | 0.664 |
|  | *Boops boops* | (Linnaeus, 1758) | non commercial | vagile | 0.146 |
|  | *Diplodus puntazzo* | (Walbaum, 1792) | commercial | vagile | 0.011 |
|  | *Diplodus cervinus* | (Lowe, 1838) | commercial | vagile | 0.582 |
|  | *Diplodus vulgaris* | (Geoffroy Saint-Hilaire, 1817) | commercial | vagile | 0.538 |
|  | *Oblada melanura* | (Linnaeus, 1758) | non commercial | vagile | 0.346 |
|  | *Spondyliosoma cantharus* | (Linnaeus, 1758) | commercial | vagile | 0.003 |
|  | *Lithognathus mormyrus* | (Linnaeus, 1758) | commercial | vagile | 0.002 |
|  | *Diplodus cadenati* | (Linnaeus, 1758) | commercial | vagile | 0.414 |
|  | *Pagrus pagrus* | (Linnaeus, 1758) | commercial | vagile | 0.002 |
| Sphyraenidae | *Sphyraena viridensis* | (Cuvier, 1829) | commercial | vagile | 0.072 |
| Synodontidae | *Synodus saurus* | (Linnaeus, 1758) | bycatch | sedentary | 0.001 |
|  | *Synodus synodus* | (Spix & Agassiz, 1829) | commercial | sedentary | 0.010 |
| Tetraodontidae | *Canthigaster capistrata* | (Bloch, 1786) | non commercial | vagile | 0.012 |
|  | *Sphoeroides marmoratus* | (Lowe, 1838) | non commercial | vagile | 0.009 |
| Torpedinidae | *Torpedo marmorata* | (Risso, 1810) | bycatch | sedentary | 0.001 |
| Trachinidae | *Trachinus draco* | (Linnaeus, 1758) | commercial | sedentary | < 0.001 |
|  | *Trachinus radiatus* | (Cuvier, 1829) | commercial | sedentary | < 0.001 |
| Tripterygiidae | *Tripterygion delaisi* | (Cadenat & Blache, 1970) | non commercial | sedentary | < 0.001 |
|  |  |  |  |  |  |
